# Analysis of Factors Influencing the Precision of Body Tracking Outcomes in Industrial Gesture Control

**DOI:** 10.3390/s24185919

**Published:** 2024-09-12

**Authors:** Aleksej Weber, Markus Wilhelm, Jan Schmitt

**Affiliations:** Institute of Digital Engineering, Technical University of Applied Sciences Würzburg-Schweinfurt, Ignaz-Schön-Straße 11, 97421 Schweinfurt, Germanyjan.schmitt@thws.de (J.S.)

**Keywords:** bodytracking, Azure Kinect, gesture control, industrial environment

## Abstract

The body tracking systems on the current market offer a wide range of options for tracking the movements of objects, people, or extremities. The precision of this technology is often limited and determines its field of application. This work aimed to identify relevant technical and environmental factors that influence the performance of body tracking in industrial environments. The influence of light intensity, range of motion, speed of movement and direction of hand movement was analyzed individually and in combination. The hand movement of a test person was recorded with an Azure Kinect at a distance of 1.3 m. The joints in the center of the hand showed the highest accuracy compared to other joints. The best results were achieved at a luminous intensity of 500 lx, and movements in the x-axis direction were more precise than in the other directions. The greatest inaccuracy was found in the z-axis direction. A larger range of motion resulted in higher inaccuracy, with the lowest data scatter at a 100 mm range of motion. No significant difference was found at hand velocity of 370 mm/s, 670 mm/s and 1140 mm/s. This study emphasizes the potential of RGB-D camera technology for gesture control of industrial robots in industrial environments to increase efficiency and ease of use.

## 1. Introduction

RGB-D cameras can be compact in design, intuitive in application, cost-effective, and markerless. However, the performance of these sensing devices varies significantly due to their diversity [1]. In research contexts, the Vicon multi-camera motion capture system, is often considered the gold standard in terms of accuracy and precision [2]. Other markerless body tracking systems, such as Microsoft’s Kinect cameras, are less powerful compared to the Vicon system but are considerably more affordable, user-friendly, and easy to integrate into an existing system [2]. This is because marker-based body tracking often requires multiple recording sensors and specialized laboratories for measurements. These factors frequently determine the application scope of this technology. In recent years, there has been an increased focus in the scientific community on various approaches to analyze and optimize the precision and accuracy of RGB-D cameras. This includes environmental analyses concerning potential disturbances that could affect the cameras’ performance. In the context of this work, potential disturbances in the application of an RGB-D camera for gesture control of an industrial robot are examined. These disturbances have not been sufficiently studied scientifically for the industrial environment. The goal is to identify these disturbances and investigate their potential impact, both individually and in combination, on accuracy and precision in an experiment. Based on the results, recommendations will be made regarding the parameters to be set in order to minimize the influence of the examined disturbances. For this purpose, conditions typical of an industrial environment are examined, with a focus on ambient lighting, range of motion, and velocities. This analysis is conducted for different movement directions to assess the quality of tracking depending on the direction. Based on a “Design of Experiments” (DoE), a real person is captured using a depth camera, with hand movements being precisely controlled by a linear guide. The captured motion data are then statistically analyzed. Additionally, the suitability of the technology for controlling an industrial robot in an industrial environment will be critically discussed.

## 2. Literature

In the existing literature, a multitude of disturbances have been identified that can negatively impact the accuracy and precision of an RGB-D camera. In the context of this work, disturbances refer to all external factors present in the camera’s environment. In contrast to this, the recording process itself is not analyzed, as is the case with Büker et al. [3]. The overview also does not concentrate on work that examines the fundamental accuracy of the system as, for example, Kurillo et al. [4]. The focus is on the environmental variables, in particular. The following literature review presents relevant scientific works (Table 1), examining:

- Research area;
- Used RGB-D camera;
- Type of external influencing factors;
- Type of measurement.

Initially, the reviewed works are categorized based on the research area into Medicine, Technology, and Sports. Furthermore, a differentiation of recording devices is made, distinguishing between Kinect v1 (Kv1), Kinect v2 (Kv2), Azure Kinect (AK), and Others. The type of external influencing factors is classified into five categories: Camera Position, Human Position, Light Influence, Technical Progress, Velocity and Others. Camera and human positioning refer to the precise placement and orientation of the instance. Regarding light influence, all works investigating the impact of light on the performance of an RGB camera are summarized. Technical progress provides insights into whether newer technologies were compared. The term Velocity explains the analyzed speeds in the work. Finally, the “Others” category encompasses various aspects that do not fit into the aforementioned categories.

The works of Pfister et al. [5], Ťupa et al. [6], Xu et al. [7], Albert et al. [2], and Yeung et al. [15] examine the gait of participants with the objective of identifying gait characteristics and investigating the suitability of RGB-D cameras for use in the medical field. In their study, Pfister et al. [5] studied 20 participants walking and jogging at speeds of 1.34 m/s, 2.01 m/s, and 2.45 m/s on a treadmill. The experiment showed that the best results were achieved at the lowest speed. In their study, Ťupa et al. [6] examined the gait of 18 individuals with Parkinson’s disease, 18 healthy controls, and 15 students while they walked forward and backward. The highest accuracy was achieved at a speed of 0.73 m/s. As demonstrated by Xu et al. [7], the degree of measurement error varies contingent on the speed and the variable being measured. To illustrate, the investigated speeds exert no notable influence on the accuracy of step duration and step length (defined as the interval between two consecutive heel strikes of the same foot). Nevertheless, there is a notable impact of speed on the accuracy of step width recognition. To this end, the researchers examined 20 test subjects on a treadmill with the RGD-D cameras positioned in front of the subjects at speeds of 0.85 m/s, 1.07 m/s, and 1.30 m/s. In a further development of the work presented in Xu et al. [7], Albert et al. [2] evaluated the performance of Kv2 and AK. The experiment was conducted with five participants at treadmill speeds of 0.85 m/s, 1.07 m/s, and 1.30 m/s in a frontal position. The results demonstrated that the enhanced hardware and motion tracking algorithm of the AK resulted in markedly higher accuracy than the Kv2. However, both versions exhibited a greater measurement error for the extremities in comparison to the center of the body, due to the higher movement speeds and greater range of motion of the extremities. Yeung et al. [15] conducted an analysis of the performance of treadmill gait pattern recognition from five camera positions (0°, 22.5°, 45°, 67°, 90°) and three walking speeds (0.83 m/s, 1.22 m/s, 1.64 m/s). The camera was positioned at a height of 1.2 m and a distance of 2.4 m from the subject. No significant difference in accuracy was observed at the speeds tested. In general, the AK demonstrated superior tracking accuracy in comparison to Kv2, with the exception of the 0° camera position.

The works of Otte et al. [8], Abbondanza et al. [9], Mobini et al. [10], Shanyu et al. [13], Wasenmüller and Stricker [12], Faity et al. [16], and Novo et al. [17] investigate the performance of RGB-D cameras under different external conditions. In their study, Otte et al. [8] recorded and analyzed the performance of 19 participants undertaking six different movement tasks. The findings indicated that head tracking exhibited superior accuracy compared to foot tracking. Furthermore, the accuracy of the system was found to vary according to the type of movement performed. Specifically, vertical movements of the head, shoulder, hand, and wrist exhibited higher accuracy. It was observed that an increase in the range of motion resulted in a corresponding increase in inaccuracy for the same movement. In order to ascertain the accuracy of measurements taken from a mannequin, Abbondanza et al. [9] conducted experiments under a variety of conditions, including different positions, lighting (on/off), and the presence/absence of clothing and covered limbs. The findings indicated that the optimal positioning of the RGB-D camera is such that it does not obscure any body parts. Furthermore, the study revealed that lighting conditions had no significant impact on the performance of the RGB-D camera. Furthermore, the results demonstrated that a clothed mannequin exhibited superior accuracy compared to an unclothed one. Additionally, the greatest measurement inaccuracy was observed at an angle of 95° to 135° between the person and the camera. In their study, Mobini et al. [10] examined the performance of ten vertical and ten diagonal hand movements at varying speeds. The movements were classified as either <3 m/s or >3 m/s for vertical movements and either <4 m/s or >4 m/s for diagonal movements. The optimal results for RGB-D cameras were observed for movements at the lowest speed. Furthermore, superior performance was noted at higher speeds for diagonal motion in comparison to vertical motion. In a separate study, Kawaguchi et al. [11] recorded the body and hand movements of ten participants for eight seconds and compared the data with a reference system. The experiment demonstrated that the measurement error increased in conjunction with the velocity of movement, even at relatively low speeds when the hand was in close proximity to the body. In their study, Wasenmüller and Stricker [12] conducted a comprehensive comparison of Kv1 and Kv2. Their findings revealed that while Kv2 remained consistent with increasing camera distance, precision exhibited a decline. It was demonstrated that surfaces with lower reflectivity, such as dark clothing, exhibit less reliable depth estimation compared to those with higher reflectivity. In the study conducted by Faity et al. [16], 26 participants were instructed to perform seated reaching tasks while holding a dumbbell, in order to simulate the movement pattern of a partially paralyzed individual. The findings indicated that Kv2 exhibited moderate accuracy in the assessment of hand movement range, movement time, and average speed. However, the detection of elbow and shoulder range of motion, time to peak velocity, and distance traveled exhibited low to moderate reliability. In their study, Novo et al. [17] examined the tracking of a mannequin in a two-armed lifting and lowering motion in both standing and sitting positions. The study demonstrated that the optimal positioning of Kv1 was directly in front of and above the mannequin at a distance of 1.30 m. Furthermore, an enhancement in accuracy was noted as the depth contrast between the mannequin and the surrounding environment increased. No significant difference was observed between the slow (0.2 m/s) and fast (0.4 m/s) motion trials. In their study, Shanyu et al. [13] employed the Kv1 technique to construct a three-dimensional body model. The objective was to identify the optimal parameters for posture screening. The findings indicated that the accuracy of Kv1 diminished with augmented light intensity. Accordingly, an optimal light intensity of 80 lx was proposed. Additionally, the authors advised that the RGB-D camera should be positioned at chest height and at a distance of 1.30 m from the subject. The work of Tölgyessy et al. [14] evaluates the skeleton tracking accuracy and precision of Kv1, Kv2, and AK across different distances and sensor modes. In their study, a human-sized figurine was placed on a robotic manipulator, and joint detection accuracy was measured at distances of up to 3.6 m. The results indicated that the AK outperformed its predecessors. The AK showed superior performance compared to Kv2 and Kv1, especially in tracking body joints closer to the center of the body. Tracking of extremities showed higher error rates due to the increased range of motion.Bertram et al. [18] evaluated the accuracy and repeatability of the AK for clinical measurement of motor function in 30 healthy adults. It compared AK’s performance to the previous Kv2 and a marker-based motion capture system (Qualisys). In summary, AK showed the best performance in dynamic tasks and upper body movements, but weaknesses in stationary tasks and tracking movements of the feet and ankles. Büker et al. [19] investigate the impact of varying illumination conditions on the body tracking performance of the Azure Kinect DK device, with the aim of ensuring the reliability of results in research involving human subjects. Two experiments were conducted. The first experiment employed four distinct lighting conditions, while the second experiment involved repeated measurements under similar lighting conditions. The researchers discovered that maintaining consistent light conditions resulted in comparable outcomes with minimal discrepancies, with a maximum of a 0.06 mm difference. However, varying light conditions introduced inconsistencies, with an error range up to 0.35 mm. The use of supplementary infrared light was identified as a particularly detrimental factor affecting the accuracy of the tracking process.

In conclusion, the majority of relevant literature is of a medical nature. Nevertheless, the existing literature provides valuable insights into factors that may subsequently influence performance. In general, the literature demonstrates that the performance of RGB-D cameras improves with technological advancement [2,12,15]. The positioning of RGB-D cameras should be chosen to avoid obscuring or overlapping body parts [9,11]. It has also been shown that the camera angle between the subject and the camera can have a significant effect on the result, emphasizing the need to position the RGB-D camera directly in front of the subject [9,15,17]. The distance between the subject and the RGB-D camera should not be too great, as accuracy decreases with distance [12]. For Kv1, the optimal distance is 1.30 m [12,13]. The performance of RGB-D cameras is also affected by clothing, clothing color, and contrast with the environment [9,12,17]. Abbondanza et al. [9] found that Kv2 results were not affected by lighting conditions, whether on or off. However, Shanyu et al. [13] found that the accuracy of Kv1 decreased with increasing light intensity and recommended an optimal light intensity of 80 lx. Regarding the speed-related factor, the scientific community has reached different conclusions. While Pfister et al. [5], Mobini et al. [10], Kawaguchi et al. [11], and Albert et al. [2] found the best performance of RGB-D cameras at the lowest tested speed, Xu et al. [7], Yeung et al. [15], and Novo et al. [17] found no significant difference at the tested speeds. The different results may be due to the choice of measurement parameter. Otte et al. [8] and Albert et al. [2] showed that the measurement error is higher for the lower and upper extremities. This is due to the greater range of motion and faster movement of the extremities compared to the rest of the body. Mobini et al. [10] also showed that at higher speeds the accuracy of Kv1 is higher for diagonal hand movements than for vertical hand movements. However, Otte et al. [8] found that Kv2 had the highest accuracy for vertical movements.

## 3. Method and Experimental Setup

The following sections describe the procedure, structure and sequence for the experiment to be carried out.

### 3.1. Method

In order to determine possible interactions between the selected parameters, the test is carried out according to the full factorial test principle. All factor combinations are analyzed for possible dependencies. The experimental effort na results from the factor sum n_*f*_ and the number of steps n_*s*_ (Equation (1)) [20].

(1) $$n_{a}=n_{s}^{n_{f}}$$

A total of four factors n_*f*_ = 4 are defined, each with three levels n_*s*_ = 3. According to the full factor plan, this results in 81 condition modifications, which are analyzed in the experiment.

### 3.2. Parameters

In the beginning, the parameters developed are classified, and evaluated individually and relevant measurement variables are defined for the statistical test plan. They are categorized into three groups: controlled, unconsidered and independent variables. Controlled variables are kept constant in the experiment so that they have no influence on the result of the independent parameters. Influencing variables that are not taken into account are not integrated into the experiment, as they are not considered relevant and would exceed the scope of a statistical experimental design. The independent variables are analyzed in the statistical test plan with regard to their individual and interaction effects on the accuracy and precision of an RGB camera. In Table 2, the results from the literature review and other external disturbance variables that can occur in an industrial environment are sorted according to the reference variables human, environment and RGB-D camera and classified according to their evaluation.

The influencing variables of sound, mechanical oscillations, pressure, vapors, air movement, machine waste heat and gases are not taken into account in the experiment. The entire test is carried out with an AK from Microsoft. It is assumed that the test results could also apply to similar RGB-D cameras. The AK used is checked for possible damage and functionality before the start of the experiment. Temperature and humidity can vary greatly in a production hall, for example, due to machine waste heat, type of production, time of year, etc. In the experiment, the temperature and humidity parameters are kept constant with regard to the technical specifications of the AK from [21]. The quantity of dirt particles can fluctuate to varying degrees depending on the product area. The decisive factors here are the intensity of the contamination and the duration of exposure. Nonetheless, measures such as the skillful positioning of the RGB-D camera or regular cleaning (inclusion in work instructions or cleaning schedule) mean that the disturbance variable of contamination can be neglected for this experiment. In the experiment, the RGB-D camera is thoroughly cleaned before use. Similar to the degree of pollution, the vibration factor can be almost eliminated by measures such as placing the AK on an anti-vibration mat. To prevent the results of the experiment from being falsified by other persons, only the subject may be in the recording zone during the test. In order to keep the depth contrast between the subject and the environment constant, the subject will wear dark, long clothing during the entire experiment [9,12]. As recommended in the literature, the AK positioning is at a distance of 1.3 m in front of the subject [17] and an angle between the camera and subject of 0° [9,15,17]. The camera height is 1.8 m, which corresponds to a position slightly above the subject. The vertical inclination is 30° downwards [17]. As stated in the literature review, the result of an RGB-D camera can also depend on the direction of movement [8,10]. In the experiment, three degrees of freedom of the translational hand movement direction are, therefore, analyzed separately for accuracy and precision. The hand movement range is defined according to DIN EN 614-1:2006-07 [22]. The standard specifies that the range should be carried out according to the fifth percentile or lower. For this purpose, the distances of 0.5 m, 0.25 m and 0.125 m are selected for the test, which are within the range of the fifth percentile of the woman according to DIN 33402-2:2020-12 [23]. The reason for the choice of distance is the movement range-dependent accuracy found in the literature review [2,8]. In conjunction with the analysis of the hand movement direction and the hand movement range, the hand movement speed is also analyzed. As noted in the literature review, there are large variations in performance in terms of both speed and the choice of measurement size [2,5,7,10,11,15,17]. For the purposes of this study, the movement of the right hand is to be tracked. Based on the work of Grosskopf [24], 1.14 m/s for fast, 0.67 m/s for normal and 0.37 m/s for slow hand movements were selected for the hand movement speeds. Furthermore, a hand movement consists of a positive and a negative acceleration. For the experiment, it is assumed that both accelerations are constant regardless of the range and speed of movement. For this purpose, a positive or negative acceleration of 10 m/s^2^ is defined, which is also used in the work by Mobini et al. [10]. In order to investigate a possible influence of the light intensity on the results of the AK, the light intensities for the experiment are determined according to the recommendation of the “Rules for Workplaces” (ASR) for an assembly workstation. The ASR specifies a luminous intensity of 200 lx for coarse assembly activities, 300 lx for medium-fine and 500 lx for fine assembly activities [25]. An examination of 80 lx, which Kawaguchi et al. [11] recommend in their work, is not examined in this study. The reason for this is the lack of lighting, which can be assumed to be unlikely in a production environment. Furthermore, care was taken to ensure that no reflections from objects or light could occur within the test setup. As the individual height of different test subjects could lead to different test results, only one test subject should be used for the experiment. Table 3 summarizes the selected parameters to be investigated in the experiment with the respective number of stages and stage designation.

### 3.3. Experiment Setup

**Figure 1:**
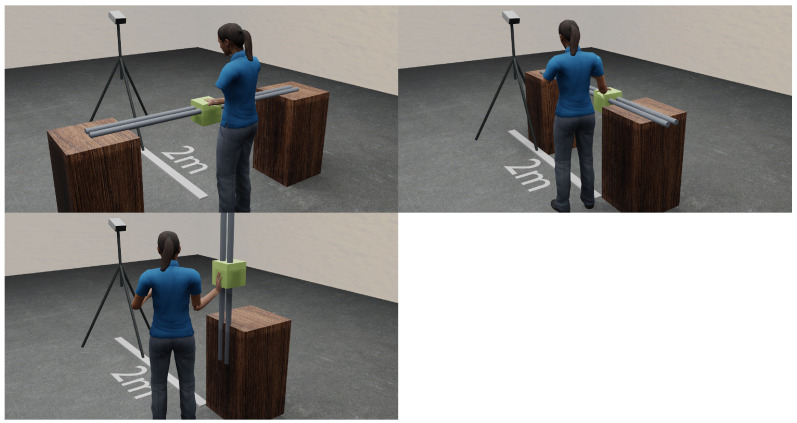
Experimental setups in x-direction (**top left**), y-direction (**top right**) and z-direction (**bottom**).

A test subject is positioned 1.3 m frontally in front of an AK. The angle between the subject and the RGB-D camera is kept constant during the test. The AK is aligned to a height of 1.80 m and a downward inclination of 30°. A linear guide (LG) with a movable device, on which the hand is later placed, is positioned directly in front of the subject. The palm of the hand is kept open and the fingers extended during the test. To ensure uniform hand positioning, a negative impression of the subject’s hand was created using commercially available mounting foam and colored black to minimize reflections. The height of the upper edge of the LG is 1.20 m. The experiment is conducted with one subject (gender: male; age: 35 years; height: 1.73; weight: 76 kg). In order to realize the three directions of movement with one LG, the test setup is modified in the course of the experiment. For this purpose, the LG is reorganized according to the x-, y- and z-axis. The relative distances, as described above, are retained. The camera position remains unchanged. Figure 1 shows the test setup with the respective positioning of the LG depending on the direction of axis movement.

It should also be noted that when realizing the setup in the z-direction, the palm is placed vertically on the side of the support device (see Figure 1, bottom). In this case, the back of the hand points towards the subject. In contrast, in other configurations the hand rests horizontally on the resting device. The experiment takes place in a laboratory that allows precise adjustment of the lighting conditions. The light color is kept the same for all experiments. To ensure exact light control, the laboratory is completely darkened in advance. The light intensity is determined using a luxmeter.

## 4. Material and Data Analysis

The following sections describe the software and hardware utilized, as well as the data analysis methodology employed.

### 4.1. Hard- and Software

A toothed belt axis (ZLW-20120 41425050 001 001, igus GmbH, cologne, Germany) with a stroke of 1 m and a repeat accuracy of 0.1 mm is used in the test. This linear axis is driven by an EC motor (MOT-EC-86-C-K-A, igus GmbH, cologne, Germany) with a 20:1 gearbox (GEA-60-20-90-ST, igus GmbH, cologne, Germany). The motor and linear axis are controlled using a drylin® D1 motor controller for stepper (igus GmbH, cologne, Germany), DC and EC/BLDC motors. The wiring and configuration of the components are carried out according to the specifications of the drive D1, ST-DC-EC/BLDC-Motor Control System Manual (V3.0.1). Two 24 V power sources are connected in series to supply power to the EC motor in order to realize a total voltage of 48 V. The control logic is supplied by a separate 24 V power source. A braking resistor of 2.8 Ω and a power of 100 watts are connected in order to intercept voltage peaks during braking processes, which can be higher than the maximum permissible supply voltage of the load circuit. The linear axis is referenced using a positively switched encoder. All test runs are carried out with an Azure Kinect. Data are captured at a frequency of 30 Hz and a depth resolution of 640 × 576 in NFOV (Narrow Field Of View) and unbinned mode. The latter enables a higher resolution and precision of the depth information, as each pixel has an independent depth measurement (Microsoft, 2022). A light spectrometer from Asensetek Inc. (model no: ALP-01, New Taipei, Taiwan) is used to check the ambient light conditions. This measuring device makes it possible to precisely quantify and analyze the light in lux [lx], which ensures that the desired lighting conditions are maintained. The entire control and data acquisition is operated by a computer equipped with an Intel^®^ Core™ i7-9700K processor (Intel Corp., Santa Clara, USA) with eight cores and a clock frequency of 3.60 GHz and 32 GB RAM. Windows 10 is used as the operating system and an Nvidia GeForce RTX 4070 Ti graphics (Nvidia Corp., Santa Clara, USA) card is available for graphics processing. The data analysis is carried out with RStudio (2023.09.1 Build 494).

### 4.2. Standard Error and Sample Size

In order to quantify the uncertainty or scatter of the measured data and to exclude humans as an additional factor, ten separate recordings of an identical movement of the right hand under constant conditions are carried out in advance. This allows the accuracy and reliability of measurements to be assessed and their effects to be taken into account. The standard error of the estimated expected value is derived from the Equation (2) [26].

(2) $$\hat{\sigma_{\bar{x}}}=\frac{\sqrt{S^{2}}}{\sqrt{n}}$$

The results show that there is an uncertainty of 1.78 mm in the x-direction, 1.15 mm in the y-direction and 1.51 mm in the z-direction. Furthermore, the standard error is very small depending on the person and can be neglected for the data analysis. The sample size is calculated using G*Power (V3.1.9.7). The selected parameters are configured in such a way that even minor influences of the independent variables can be recorded with statistical significance. In this context, it is assumed that the effect size $f^{2}$ is to be categorized as small, which is why it is assessed as 0.07. The result of this calculation shows that a sample size of 5662 is necessary to be able to make a statement with a confidence level of 95%. The test is, therefore, carried out with a sample size of 6000. The sample code provided on GitHub by Microsoft [27] is used as the basis of the code for data acquisition with the Azure Kinect. The programming is conducted in Visual Studio 2022 and is implemented in C programming language. The test procedure is essentially divided into two phases. In phase I, the test person is asked to confirm the placement of the hand with the enter key, whereupon a countdown is initialized. At the end of the countdown, 1000 frames of the calmly positioned hand are then recorded on the linear axis in order to reference the hand start position. This makes it possible to compare the relative change in the position of the hand. The reason for this is the internal coordinate system of the AK, which can vary when starting a recording. Phase II starts with a further countdown. After the countdown, the linear axis begins to move according to the set parameters. The movements of the hand are recorded in parallel with the AK until a total of 6000 frames have been recorded. A frame contains the following four joints of the right hand:

- Joint (14)—right wrist;
- Joint (15)—right hand;
- Joint (16)—fingertip middle finger right;
- Joint (17)—thumb right

and the corresponding x-, y- and z- coordinates. The data recorded for the right hand are written to CSV files.

### 4.3. Data Analysis

**Figure 2:**
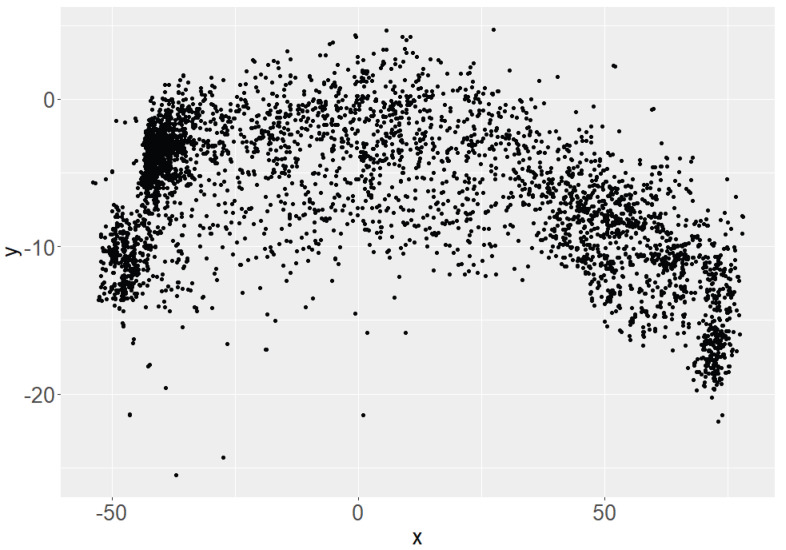
Exemplary distribution of the measured positions of joint (15) of the x- and y-axis without regression function.

**Figure 3:**
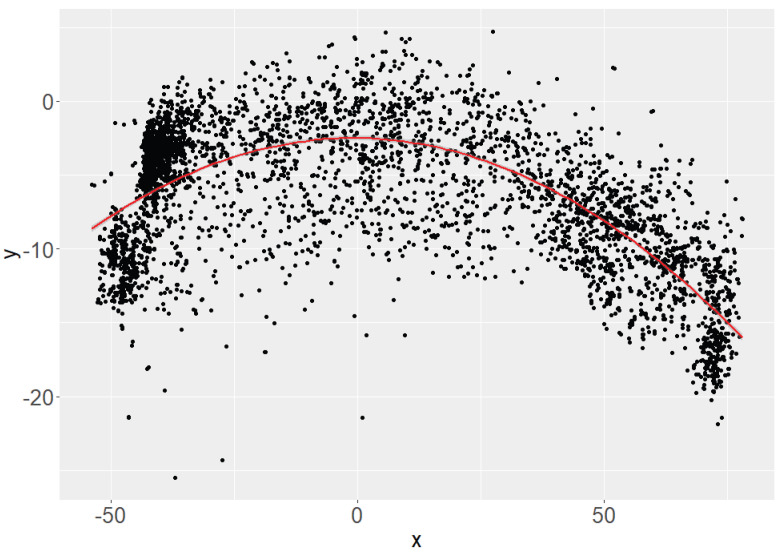
Exemplary distribution of the measured positions of joint (15) of the x- and y-axis with quadratic regression function (red curve).

When analyzing the data set, it is noticeable that the distribution of the detected data points has a parabolic shape (see Figure 2 and Figure 3). The lens curvature is assumed to be the cause of this pattern. In order to be able to make a statement about accuracy and precision, it is necessary to use a measure that is robust against outliers and can be easily interpreted. To assess the accuracy, the Mean Absolute Error (MAE) is used as the statistical measure. A regression curve is then fitted to the scatter of data points to analyze the relationship and quantify the errors.

To determine the MAE, the differences between the actual data points and the values estimated by the regression function are calculated. These differences are then taken in absolute terms to ensure that both positive and negative deviations are weighted equally. Averaging these absolute differences gives the MAE. In mathematical terms, the MAE (Equation (3)) is calculated as the sum of the amounts of the differences between the actual and estimated values divided by the number of data points [28].

(3) $$MAE=\frac{1}{n}\sum_{i=1}^{n}\left|y_{i}-{\hat{y}}_{i}\right|$$

To make it easier to compare the data, the calculated MAEs are labeled MAE_1 and MAE_2. These designations are derived from the resulting movement ranges, which depend on the direction of hand movement. For example, if a hand movement takes place in the x-axis direction, one MAE is determined for the y-axis deviation and one MAE for the z-axis deviation. In contrast, for a hand movement in the z-axis direction, one MAE is calculated for the x-axis deviation and one MAE for the y-axis deviation. Table 4 shows the respective assignment of the MAE values depending on the direction of movement.

## 5. Results

**Figure 4:**
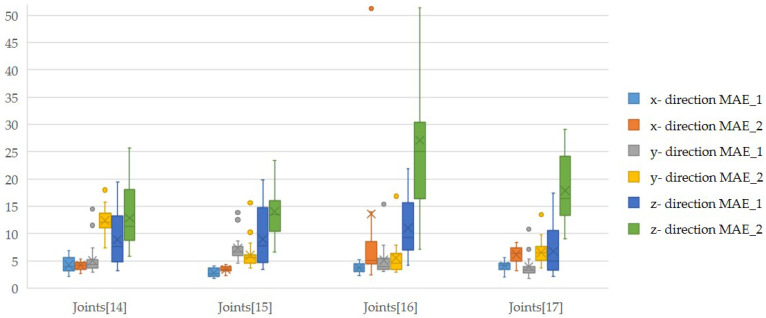
Distribution of the MAEs per direction of movement for all data sets of the joint (15)–Joint (17).

The results show that the joint (15) (right hand) has the lowest variance for all the test series carried out. If the linear guide is aligned in the X direction, the Y and Z positions should ideally remain stable, as the joints to be observed are firmly connected to the guide. In this arrangement, movement is only possible in the X direction, while the other axes should ideally show no deviations from the initial value. The same principle applies if the guide is aligned along the Y or Z axis; only the movement along the aligned axis should take place, while the values for the others should remain constant. Based on the average of the MAEs and the corresponding standard deviations (SD) for the axes, which should remain constant, it is found that the average error is the smallest when the movement is in the X direction, indicating better detection. Conversely, the average of the errors is highest for movements in the Z direction, which indicates lower detection accuracy. This trend is visually confirmed by the box plots, which clearly show the differences in accuracy between the different directions of movement. These results are also supported by the subsequent analyses with different parameters. Overall, the joint (15) exhibits the highest precision and accuracy of the joints tested. The highest inaccuracy is found at joint (16) (fingertip on the right) (see Figure 4).

**Figure 5:**
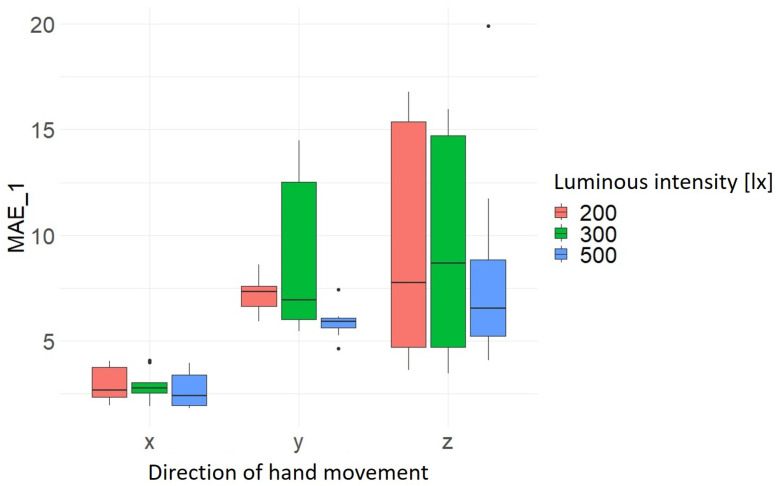
Distribution MAE_1 as a function of hand movement direction and light intensity [lx].

**Figure 6:**
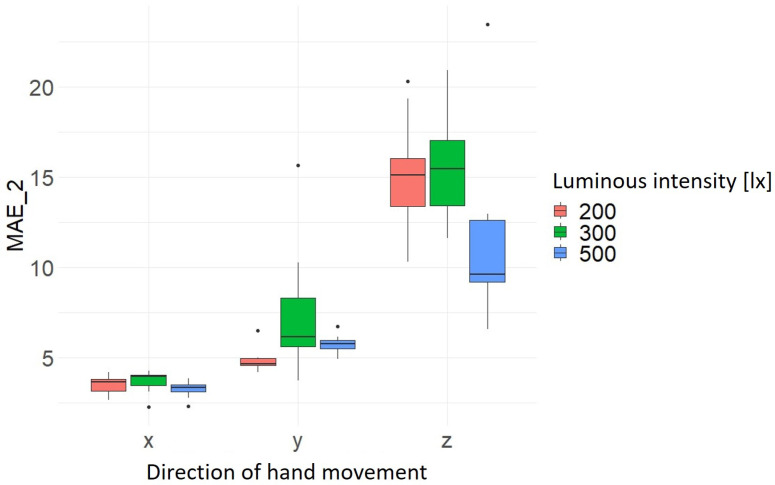
Distribution MAE_2 as a function of hand movement direction and light intensity [lx].

In the following, the influence of the independent variables is evaluated using joint (15). For this purpose, boxplots are created to visualize the distribution of the values of MAE_1 and MAE_2 in relation to the hand movement direction as a function of light intensity, hand movement range and hand movement velocity. The present results show that the highest measured accuracy and precision of 2.64 mm ± 0.84 mm is achieved at a light intensity of 500 lx. Furthermore, it can be seen that the results for the light intensities of 200 lx and 300 lx are almost identical overall in terms of accuracy. However, the precision varies to different degrees depending on the direction of hand movement, as can be seen by comparing Figure 5 and Figure 6 and Table 5. Table 5 summarizes the results of the average values of the MAEs (mean) and standard deviation (sd) for MAE_1 and MAE_2 as a function of the hand movement and luminous intensity.

**Figure 7:**
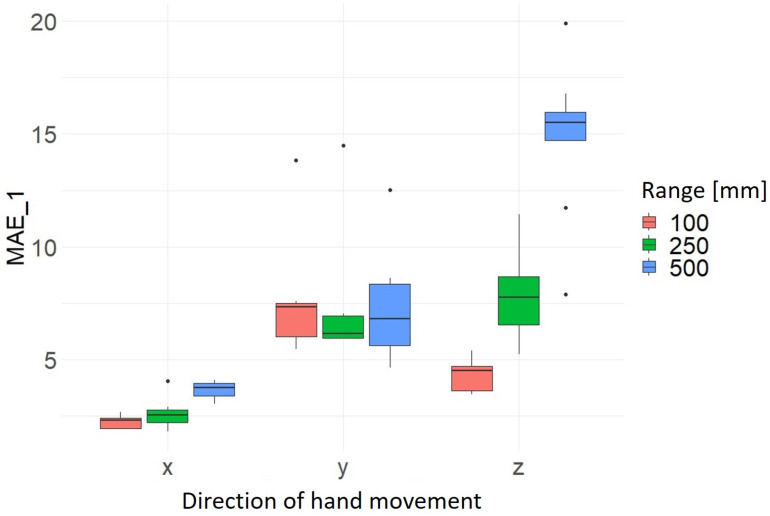
Distribution MAE_1 as a function of hand movement direction and range of hand movement [mm].

**Figure 8:**
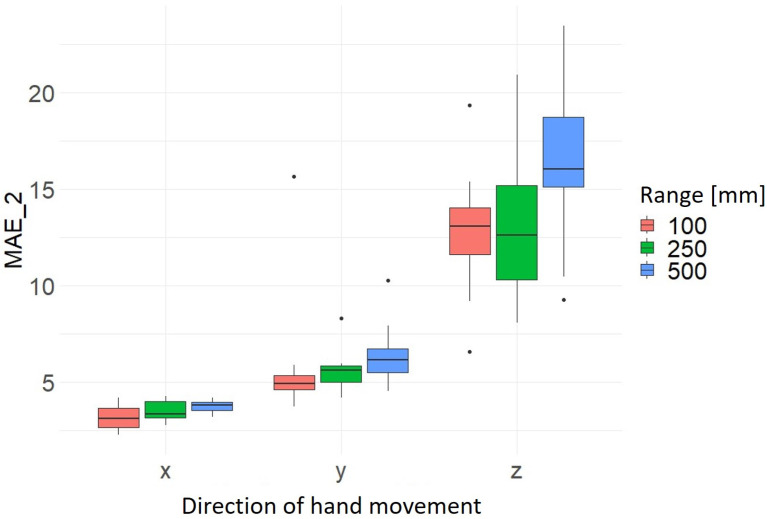
Distribution MAE_2 as a function of hand movement direction and range of hand movement [mm].

The results for the hand movement range show noticeable that in most cases the average MAEs are highest at 500 mm. This means that the deviation of the normally constant values of the axes not in motion is the greatest on average across the 10 trials for each direction and range. Thus, the highest accuracy and precision for a movement range of 100 mm is measured at 2.24 mm ± 0.30 mm. It can also be seen that the results for the x-axis direction of movement are the most precise. As previously stated, the greatest deviation is recorded in the z-axis movement direction for a movement range of 500 mm at 16.20 mm ± 0.40 mm (see Figure 7 and Figure 8 and Table 6). The calculated average values and the corresponding standard deviation for MAE_1 and MAE_2 of the hand movement directions and hand movement ranges investigated are summarized in Table 6.

**Figure 9:**
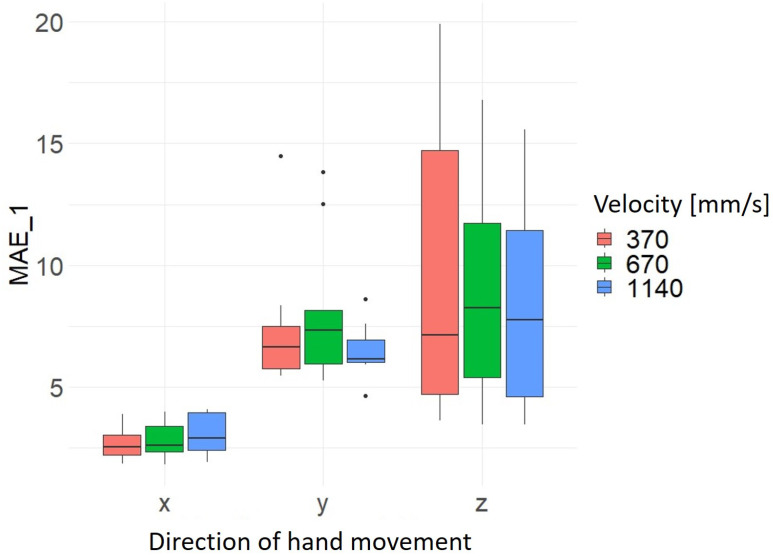
Distribution MAE_1 as a function of hand movement direction and velocity of hand movement [mm/s].

**Figure 10:**
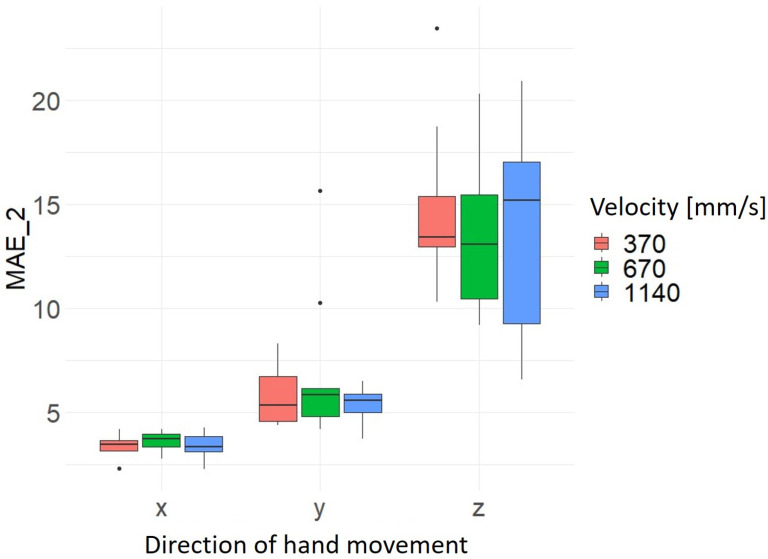
Distribution MAE_2 as a function of hand movement direction and velocity of hand movement [mm/s].

With regard to hand movement velocity, there is no clear pattern in terms of speed-dependent precision or accuracy. However, the results confirm the finding already mentioned above that the hand movements in the x-axis direction have the highest precision and accuracy and in the z-axis direction the lowest precision and accuracy with regard to the hand movement direction (see Figure 9 and Figure 10). However, the significantly lower scatter of the data sets for the hand movement in the x- and y-axis direction is clearer here. The results of the average values and standard deviation for MAE_1 and MAE_2 as a function of hand movement direction and hand movement velocity are summarized in Table 7.

## 6. Discussion

The aim of the work was to analyze and optimize the precision and accuracy of an RGB-D camera, particularly in the context of gesture control of an industrial robot. To this end, potential external disturbance variables were identified and evaluated using existing scientific work. The selected parameters, including light intensity, hand movement direction, hand movement range and hand movement velocity, were worked out and the relevant factor levels were determined at the same time. The test procedure was based on a full factorial test principle, resulting in a total of 81 condition changes. The test setup consisted of positioning a subject in front of an RGB-D camera. A linear axis was used to implement the defined hand movement in order to make the hand movement as controlled and identical as possible. Accordingly, the environmental conditions characteristic of an industrial setting were replicated in a laboratory setting. However, a subsequent investigation in a real-world setting, utilizing the findings from this study, would be essential to validate the parameters identified. The results show that the light intensity has a significant influence on the accuracy and precision of the hand movement with the RGB-D camera. Sufficient light intensity, especially at 500 lx, is recommended to achieve more accurate recognition. This finding differs from the results of Abbondanza et al. [9], who found no difference in performance between light on and light off, and the findings of Shanyu et al. [13], who found decreasing accuracy for the Kinect v1 at higher light intensities. These differences may be due to the different technical specifications of the cameras. The influence of hand movement range on accuracy suggests that the precision of hand movement detection decreases as the movement range increases. This is particularly important in applications where large ranges of movement are required. This finding confirms the results of Abbondanza et al. [9] and Otte et al. [8], who also found higher inaccuracy at larger ranges of motion. In this study, hand velocity did not show a clear influence on the accuracy of the hand movement. This result is supported by the work of Xu et al. [7], Yeung et al. [15] and Novo et al. [17]. However, this differs from the results of Pfister et al. [5], Mobini et al. [10],Kawaguchi et al. [11] and Albert et al. [2], which found an increase in measurement error with increasing speed. It is debatable whether the RGB-D camera is less sensitive to hand speed, or whether the velocities chosen in this experiment were not sufficient to detect significant differences. To make a precise statement about the influence of the direction of hand movement on accuracy and precision, each direction of hand movement was considered separately in this study. This showed that more accurate results tended to be obtained when moving in the x-direction. The reason for this could be the positioning of the camera, as there was virtually no overlap of joints during x-axis movement. In particular, the z-axis movement has the most joint overlap, which could explain the poorer result. It should also be noted that each condition specification was recorded once. This could introduce unwanted singular disturbances into the measurement, which in turn, could affect the result. For example, strong vibrations during a test could cause such singular disturbances. However, it is assumed that one-off disturbances would not have a significant effect on a recording of 6000 frames.

## 7. Conclusions

This research contributes to understanding the applicability of RGB-D camera technology for gesture control in industrial environments, particularly in the context of controlling industrial robots. The identified factors influencing the accuracy and precision of hand movements provide valuable insights for the further development and optimization of this technology. Specifically, the recommendation for sufficient light intensity around 500 lx and the consideration of hand movement range is particularly relevant for practical applications. The analysis of hand movement direction underscores the importance of optimal camera positioning. The results for movements in the x-direction highlight that precise spatial positioning of the camera is essential in industrial environments. For future use, it is recommended that the technical parameters of RGB-D camera systems be carefully aligned with the specific requirements of each application. Further studies focusing on real industrial scenarios would strengthen the validity of these findings. Overall, this work suggests that RGB-D camera technology holds significant potential for enhancing gesture control in industrial robotics. By implementing and optimizing the technology with attention to the identified factors, it could further improve efficiency, precision, and user-friendliness in industrial processes.

## Figures and Tables

**Table 1:** Literature overview.

Authors	Year	Research Area	RGB-D Camera	analyzed Factor	Target
Pfister et al. [5]	2014	Medicine	Kv1, others	Velocity	Hip and knee flexion, knee extension
Ťupa et al. [6]	2015	Medicine	Kv1	Velocity	Ten skeletal points
Xu et al. [7]	2015	Medicine	Kv1, others	Velocity	Pelvis, thighs, lower legs and feet
Otte et al. [8]	2016	Medicine	Kv2, others	Others	Full-body kinematics
Abbondanza et al. [9]	2017	Engineering	Kv2, others	Camera position, human position, light condition	Lower and upper extremities, spine
Mobini et al. [10]	2017	Medicine	Kv1, others	Velocity	right hand
Kawaguchi et al. [11]	2017	Sports	Kv2, others	Velocity	right hand, full-body
Wasenmüller and Stricker [12]	2017	Engineering	Kv1,Kv2	Camera position, others	Pixel and measurement errors, different colours
Shanyu et al. [13]	2019	Medicine	Kv1, others	Camera position, light condition	Full-body
Albert et al. [2]	2020	Medicine	Kv2, AK, others	Technical progress, velocity	39 markers distributed across the entire body
Tölgyessy et al. [14]	2021	Engineering	Kv1, Kv2, AK	Technical progress, reliabilty movements	Full-body
Yeung et al. [15]	2021	Medicine	Kv2, AK, others	human position, technical progress, velocity	Joint angle measurement of the hip, knee and ankle
Faity et al. [16]	2022	Medicine	Kv2	Velocity	Upper body
Novo et al. [17]	2022	Engineering	Kv1	Camera position, human position, velocity	Shoulder and wrist joints
Bertram et al. [18]	2023	Medicine	Kv2, AK, others	Velocity, movement range, technical progress	Full-body
Büker et al. [19]	2024	Medicine	AK	Light condition	Full-body

**Table 2:** Overview of possible disturbance variables.

	Reference Unit		
Parameters	Human	Environment	RGB-D Camera
Clothes	C		
Hand movement velocity	DoE		
Hand movement direction	DoE		
Hand movement range	DoE		
Other persons	C		
Orientation	C		C
Position	C		C
Manufacturer			C
Damage			C
Body height	C		
Hand acceleration	C		
Pollution			C
Temperature		C	
Vibrations		C	
Sound		N	
Mech. oscillations		N	
Luminous intensity		DOE	
Contrast between human and environment	C	C	
Dirt particles		C	
Reflections		C	
Vapours		N	
Air movement		N	
Humidity		C	
Machine waste heat		N	
Pressure		N	
Gases		N	

C = controlled; N = not included; DoE = independent variable

**Table 3:** Independent variables.

Factor	Number of Stages	Levels
Luminous intensity [lx]	3	200, 300, 500
Hand movement velocity [m/s]	3	0.37, 0.67, 1.14
Hand movement direction [u.a.]	3	x-, y-, z-direction
Hand movement range [m]	3	0.125, 0.25, 0.5

**Table 4:** Overview of MAE designations.

Direction of Movement	MAE_1	MAE_2
x-axis direction	MAE_y	MAE_z
y-axis direction	MAE_x	MAE_z
z-axis direction	MAE_x	MAE_y

**Table 5:** Average and standard deviation of MAE_1 and MAE_2 by hand movement direction and luminous intensity.

Direction of Hand Movement	Luminous Intensity [lx]	Mean_MAE_1	Sd_MAE_1	Mean_MAE_2	Sd_MAE_2
x	200	2.97	0.80	3.51	0.56
x	300	2.90	0.72	3.69	0.64
x	500	2.64	0.84	3.24	0.45
y	200	7.23	0.84	3.24	0.45
y	300	8.93	3.64	7.55	3.65
y	500	5.87	0.75	5.77	0.51
z	200	9.22	5.25	15.10	3.15
z	300	9.45	5.14	15.70	2.90
z	500	8.23	5.00	11.40	4.95

**Table 6:** Average and standard deviation of MAE_1 and MAE_2 by hand movement direction and hand movement range.

Direction of Hand Movement	Range [mm]	Mean_MAE_1	Sd_MAE_1	Mean_MAE_2	Sd_MAE_2
x	100	2.24	0.30	3.18	0.67
x	250	2.60	0.66	3.49	0.55
x	500	3.68	0.35	3.77	0.31
y	100	7.45	2.54	6.03	3.65
y	250	7.18	2.78	5.66	1.17
y	500	7.40	2.39	6.52	1.73
z	100	4.28	0.67	12.80	3.61
z	250	7.79	1.81	13.20	3.88
z	500	14.80	3.35	16.20	4.40

**Table 7:** Average and standard deviation of MAE_1 and MAE_2 by hand movement direction and hand velocity.

Direction of Hand Movement	Velocity [mm/s]	Mean_MAE_1	Sd_MAE_1	Mean_MAE_2	Sd_MAE_2
x	370	2.68	0.72	3.42	0.56
x	670	2.80	0.76	3.60	0.49
x	1140	3.03	0.87	3.43	0.68
y	370	7.42	2.82	5.81	1.52
y	670	8.08	3.05	6.95	3.72
y	1140	6.53	1.14	5.45	0.81
z	370	9.41	5.84	15.00	3.91
z	670	9.06	4.82	13.40	3.55
z	1140	8.44	4.69	13.80	5.11

## Data Availability

The data presented in this study are available in the article.

## Abbreviations

The following abbreviations are used in this manuscript:

AK	Microsoft Azure Kinect
EC motor	electronically commutated motor
Kv1	Microsoft Kinect Version 1
Kv2	Microsoft Kinect Version 2
LG	Linear guide
MAE	Mean Absolute Error
RGB-D camera	Red Green Blue-Depth camera

## References

[1] do Carmo Vilas-Boas M., Choupina H.M.P., Rocha A.P., Fernandes J.M., Cunha J.P.S. (2019). Full-body motion assessment: Concurrent validation of two body tracking depth sensors versus a gold standard system during gait. J. Biomech..

[2] Albert J.A., Owolabi V., Gebel A., Brahms C.M., Granacher U., Arnrich B. (2020). Evaluation of the pose tracking performance of the azure kinect and kinect v2 for gait analysis in comparison with a gold standard: A pilot study. Sensors.

[3] Büker L., Quinten V., Hackbarth M., Hellmers S., Diekmann R., Hein A. (2023). How the processing mode influences azure kinect body tracking results. Sensors.

[4] Kurillo G., Hemingway E., Cheng M.L., Cheng L. (2022). Evaluating the accuracy of the azure kinect and kinect v2. Sensors.

[5] Pfister A., West A.M., Bronner S., Noah J.A. (2014). Comparative abilities of Microsoft Kinect and Vicon 3D motion capture for gait analysis. J. Med. Eng. Technol..

[6] Ťupa O., Procházka A., Vyšata O., Schätz M., Mareš J., Vališ M., Mařík V. (2015). Motion tracking and gait feature estimation for recognising Parkinson’s disease using MS Kinect. Biomed. Eng. Online.

[7] Xu X., McGorry R.W., Chou L.S., Lin J.H., Chang C.C. (2015). Accuracy of the Microsoft Kinect™ for measuring gait parameters during treadmill walking. Gait Posture.

[8] Otte K., Kayser B., Mansow-Model S., Verrel J., Paul F., Brandt A.U., Schmitz-Hübsch T. (2016). Accuracy and reliability of the kinect version 2 for clinical measurement of motor function. PLoS ONE.

[9] Abbondanza P., Giancola S., Sala R., Tarabini M. (2017). Accuracy of the microsoft kinect system in the identification of the body posture. Proceedings of the Wireless Mobile Communication and Healthcare: 6th International Conference, MobiHealth 2016.

[10] Mobini A., Behzadipour S., Saadat Foumani M. (2017). Hand acceleration measurement by Kinect for rehabilitation applications. Sci. Iran..

[11] Kawaguchi S., Takemura H., Mizoguchi H., Kusunoki F., Egusa R., Funaoi H., Takeda Y., Yamaguchi E., Inagaki S., Sugimoto M. Accuracy evaluation of hand motion measurement using 3D range image sensor. Proceedings of the 2017 Eleventh International Conference on Sensing Technology (ICST).

[12] Wasenmüller O., Stricker D. (2017). Comparison of kinect v1 and v2 depth images in terms of accuracy and precision. Proceedings of the Computer Vision–ACCV 2016 Workshops: ACCV 2016 International Workshops.

[13] Shanyu C., Chin L.C., Basah S.N., Azizan A.F. Development of Assessment System for Spine Curvature Angle Measurement. Proceedings of the 2019 8th International Conference on Software and Computer Applications.

[14] Tölgyessy M., Dekan M., Chovanec L. (2021). Skeleton tracking accuracy and precision evaluation of kinect v1, kinect v2, and the azure kinect. Appl. Sci..

[15] Yeung L.F., Yang Z., Cheng K.C.C., Du D., Tong R.K.Y. (2021). Effects of camera viewing angles on tracking kinematic gait patterns using Azure Kinect, Kinect v2 and Orbbec Astra Pro v2. Gait Posture.

[16] Faity G., Mottet D., Froger J. (2022). Validity and reliability of Kinect v2 for quantifying upper body kinematics during seated reaching. Sensors.

[17] Novo C.D., Boss R., Kyberd P., Biden E.N., Diaz J.E.T., Ricardo M.H. (2022). Testing the Microsoft kinect skeletal tracking accuracy under varying external factors. Medcrave Online J. Appl. Bionics Biomech..

[18] Bertram J., Krüger T., Röhling H.M., Jelusic A., Mansow-Model S., Schniepp R., Wuehr M., Otte K. (2023). Accuracy and repeatability of the Microsoft Azure Kinect for clinical measurement of motor function. PLoS ONE.

[19] Büker L., Hackbarth M., Quinten V., Hein A., Hellmers S. (2024). Towards comparable quality-assured Azure Kinect body tracking results in a study setting—Influence of light. PLoS ONE.

[20] Kleppmann W. (2020). Versuchsplanung: Produkte und Prozesse Optimieren.

[21] Microsoft Azure Kinect DK Hardware Spezifikationen, 2022. https://5.imimg.com/data5/SELLER/Doc/2022/8/XZ/NK/NI/14158318/microsoft-azure-kinect-dk-v4-mocap-vr-ar-camera-motion-capture-depth-sensor.pdf.

[22] (2009). ISO Central Secretary. Sicherheit von Maschinen—Ergonomische Gestaltungsgrundsatze: Teil 1: Begriffe und allgemeine Leitsätze.

[23] (2016). ISO Central Secretary. Ergonomie—Körpermaße des Menschen—Teil: 2 Werte.

[24] Grosskopf A. (2004). Kinematische Analyse von Ziel-und Greifbewegungen der dominanten und non-dominanten Hand bei beiden Geschlechtern. Ph.D. Thesis.

[25] (2016). ISO Central Secretary. Technische Regeln für Arbeitsstätten (2011), “ ASR A3.4 Beleuchtung”, ASR.

[26] Fahrmeir L., Künstler R., Pigeot I., Tutz G. (2007). Statistik–der Weg zur Datenanalyse.

[27] Microsoft Azure Kinect Samples. Berlin/Heidelberg, Germany. https://github.com/microsoft/Azure-Kinect-Samples.

[28] Willmott C.J., Matsuura K. (2005). Advantages of the mean absolute error (MAE) over the root mean square error (RMSE) in assessing average model performance. Clim. Res..

